# Appendicostomy: The Operation, Its Indications and Its Results

**Published:** 1908-09-12

**Authors:** J. Bernard Dawson

**Affiliations:** Late House Surgeon to St. Mary's Hospital.


### APPENDICOSTOMY: THE OPERATION, ITS INDICATIONS AND ITS RESULTS.

By J. BERNARD DAWSON, F.R.C.S., Late House Surgeon to St. Mary's Hospital.

The operation of appendicostomy, whilst unknown to the public and but little known to the medical profession, has nevertheless established itself as a surgical entity of use and benefit.

Briefly, the object of the procedure is to obtain a communication with the cæcum through which retained intestinal contents may be liberated or irrigating fluids introduced. Added to which it has the great advantage that there is no leakage or back-flow from the sinus, unless it has been dilated expressly for that purpose.

The skin incision is similar to that of appendicectomy, but made as small as compatible with manipulation. The muscular layers of the abdominal wall are split in the direction of their fibre and the peritoneum opened. The appendix is now sought, and it is at this step that difficulty may arise. The viscus must be moderately long and mobile to allow it to be brought out to the skin edge. Cases occur in which, either from adhesions, stricture, or kinking, there is not sufficient appendix to extend through the thickness of the abdominal wall without unduly interfering with the meso-appendix. In 100 autopsies performed at St. George's Hospital, however, it was found that appendicostomy would have been possible in 96.

The appendix is now brought to one angle of the wound, leaving if possible, ¼ in. above the skin level. The peritoneum is closed by interrupted suture of thin silk; the last stitch included the sero-muscular coat of the appendix. This suture must be lightly tied, otherwise the appendical vessels in the meso-appendix will be constricted.

The muscular layers are now allowed to fall back into position, where they are secured by one or two catgut sutures. The skin is closed with interrupted silkworm gut stitches, the last of which passes through the serous and muscular coats of the appendix. The tip of the appendix may now be cut off and a rubber catheter of suitable size—usually a No. 7 or No. 8—inserted and secured by a ligature. This procedure may lead to slight soiling of the wound—a factor which can be eliminated either by covering the wound previously with collodion or, with more certainty, by leaving the opening of the appendix for three or four days, when the wound will be sealed off by lymph. The exposed mucous membrane of the appendix should be secured by a few stitches to the skin edge.

The indications for the operation of appendicostomy are as follows:

1. *For Irrigation and Medication of the Colon.*—This is the greatest use to which the procedure has been put as yet. For the various forms of colitis, which it is not the purpose of this paper to discuss, thorough irrigation from cæcum to rectum with either sterile water or medicated lotions can be accomplished with ease and certainty. Six or eight pints can be introduced by means of a funnel and tube without producing undue distension and without pain. After a few wash-outs the patient automatically voids the lotion per anum as it enters per cæcum, thus preventing distension.

2. *For Obstinate and Protracted Constipation.*—After a few complete irrigations the introduction directly into the colon of either a few ounces of castor oil, ʒij. of magnesium sulphate dissolved in ℥iv. of warm water, or even of ℥vj. or ℥viij. of warm water, will usually relieve the most obstinate case of coprostasis. Compared with the much more formidable operations of ileo-sigmoidostomy or colectomy, this operation offers manifest advantages.

3. *For Drainage of the Cæcum and Relief of Abdominal Distension.*—Since it has been found possible to dilate an appendicostomy opening until it will admit of a rectal tube of ½ in. calibre, it will be seen that it is quite possible to efficiently drain away retained intestinal contents due to stricture below the cæcum.

4. *For Nutritive Purposes.*—The mucous membrane of the colon is capable of absorbing fluid in great quantity. This capacity seems to diminish from cæcum to sigmoid. Therefore nutrient or saline enemata introduced into the cæcum are more completely absorbed than when introduced per rectum. Possible uses for this method of feeding are in cases of gastric ulcer, where for some reason gastro-enterostomy is not justifiable or possible, and where complete rest is necessary for the stomach, and in cases of diffuse peritonitis when large saline enemata are indicated.

5. *For Irrigation and Medication of the Lower Ileum.*—It has been found possible both in the living and in the cadaver to pass a catheter through the ileo-cæcal valve to the ileum. It has been suggested that by this means irrigation might be carried out in typhoid and other ulcerative conditions of that region—a practice not unattended by danger from hæmorrhage or perforation.

The writer has been able to collect twenty cases in which this operation has been performed. In tabular form they are:—

| | |
|---|---|
| For ulcerative colitis ... ... ... | 10 |
| For simple chronic colitis ... ... ... | 6 |
| For protracted constipation ... ... | 1 |
| For other abdominal conditions ... ... | 3 |

In all the operation was successfully performed.

The remote results in the colitis series are as follows:—

Ulcerative Colitis.

| | |
|---|---|
| Total cases ... ... ... ... ... ... | 10 |
| Cases cured ... ... ... ... ... ... | 9 |

Simple Chronic Colitis with and without the Passage of Membranous Casts.

| | |
|---|---|
| Total cases ... ... ... ... ... ... | 6 |
| Cases cured ... ... ... ... ... ... | 3 |
| Improvement after one month ... ... | 1 |
| Apparent cure in four weeks with slight tendency to relapse twenty months later | 1 |
| Cure after six weeks, no subsequent history ... ... ... ... ... | 1 |

Briefly this is the method and scope of appendicostomy—an operation which has taken a place in surgery and which will probably be one of future development. Rightly it is to be regarded as a surgical aid to the treatment of a medical condition. It is encouraging that the much-abused vermiform appendix has been put to a practical use.

